# On the Question of the Course of the Hetero Diels–Alder Reactions Between *N*-(2,2,2-Trichloroethylidene)Carboxamides and Dicyclohexylcarbodiimide: A New Case of the Stepwise Zwitterionic Cycloaddition Process

**DOI:** 10.3390/molecules30132692

**Published:** 2025-06-21

**Authors:** Przemysław Woliński, Karolina Zawadzińska-Wrochniak, Ewa Dresler, Radomir Jasiński

**Affiliations:** 1 Department of Organic Chemistry and Technology, Cracow University of Technology, Warszawska 24, 31-155 Kraków, Poland; przemyslaw.wolinski@pk.edu.pl; 2 Radom Scientific Society, Rynek 15, 26-600 Radom, Poland; zofiazawadzinski@gmail.com; 3 Łukasiewicz Research Network—Institute of Heavy Organic Synthesis “Blachownia”, Energetyków 9, 47-225 Kędzierzyn-Koźle, Poland; ewa.dresler@icso.lukasiewicz.gov.pl

**Keywords:** Diels–Alder reaction, mechanism, zwitterions, carboxyimide, dicyclohexylcarbodiimide, DFT

## Abstract

The regioselectivity and the molecular mechanism of the Diels–Alder reactions between *N*-(2,2,2-trichloroethylidene)carboxamides and dicyclohexylcarbodiimide were explored based on the ωB97xd/6-311G(d) (PCM) calculations. It was found that the reaction course is determined by polar local interactions. It is interesting that the most favored reaction channel is realized not via classical single-step Diels–Alder mechanism, but according to the stepwise scheme with the intervention of the zwitterionic intermediate. The details of the electron density redistribution along the reaction coordinate were explained using the ELF technique.

## 1. Introduction

**Scheme 1:**
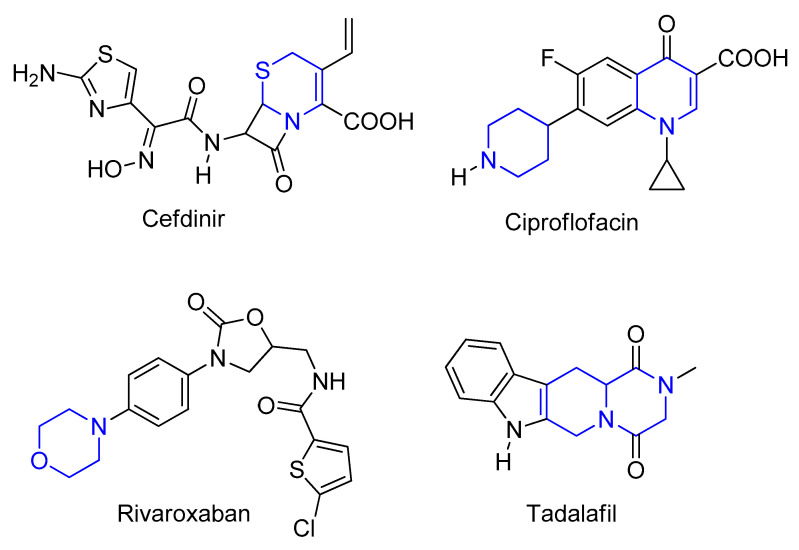
Examples of bioactive six-membered nitrogen- and oxygen-containing heterocycles.

Six-membered nitrogen- and oxygen-containing heterocycles play an important role in many areas of modern pharmacy and biotechnology: as respiratory virus inhibitors [1], anti-inflammatories [2] as well as phytochemicals and antioxidant agents [3,4] along with many other uses [5,6,7,8,9,10] (Scheme 1).

**Scheme 2:**
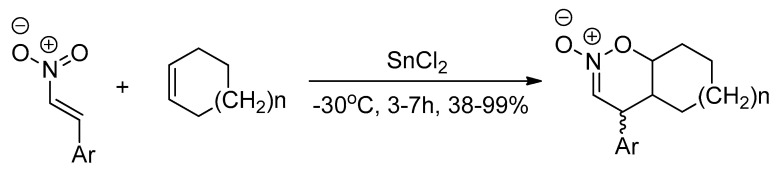
Experimentally observed course of the hetero Diels–Alder reactions between 2-aryl-1-nitroethenes and cycloalkenes.

For the preparation of this group of cyclic molecular systems many strategies are possible. However, the most universal protocol is the hetero Diels–Alder (HDA) reaction with the participation of unsaturated molecular segments of conjugated nitroalkenes [11], imines [12], vinyl aldehydes [13], nitrosocarbonyl compounds [14] and many other [15]. Many of these types of transformations require the presence of Lewis acids as catalysts [12,16,17,18]. For example, 2-aryl-1-nitroethenes react with cycloalkenes only in the presence of the SnCl_2_ (Scheme 2) [19]. Under thermal conditions this transformation is not proceeding.

**Scheme 3:**
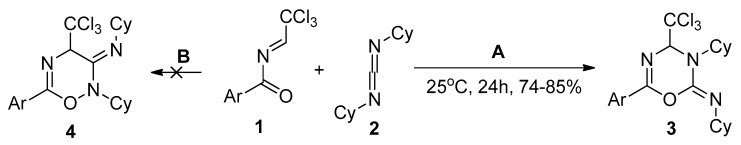
Experimentally observed course of the hetero Diels–Alder reactions between *N*-(2,2,2-trichloroethylidene)carboxamides (**1** and dicyclohexylcarbodiimide (**2**).

Very recently, Zadorozhnii and coworkers [20] described the interesting case of the HDA reaction between *N*-(2,2,2-trichloroethylidene)carboxamides (**1**) and dicyclohexylcarbodiimide (**2**). This process is realized without the presence of any catalysts and proceeded with full regioselectivity under very mild conditions (Scheme 3).

**Scheme 4:**
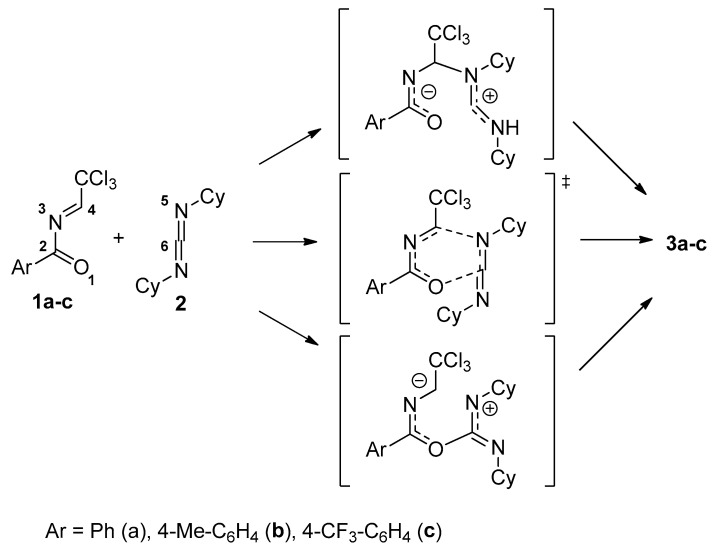
Possible molecular mechanisms for the hetero Diels–Alder reactions between *N*-(2,2,2-trichloroethylidene)carboxamides (**1** and dicyclohexylcarbodiimide (**2**).

However, the phenomenon of the high reaction regioselectivity was not explained. The molecular mechanism of the cycloaddition is not known. It should be underlined at this point that due to evidently electrophilic nature of the *N*-(2,2,2-trichloroethylidene)carboxamides **1** and nucleophilic character of the dicyclohexylcarbodiimide **2**, their cycloaddition can be realized via stepwise mechanism with the intervention of the zwitterionic intermediate [21,22]. Alternatively, the stepwise mechanism can compete with the one-step. Two types of stepwise mechanisms, determined by different regiocontrol, are theoretically possible (Scheme 4).

So, in the framework of our study, we decided to shed a light on the question of the regiodirection as well as mechanistic aspects of title processes. For this purpose, the results of the calculations based on the ωB97X-D/6-311G(d) (PCM) DFT were used. To obtain information about which of the substituents influences the reaction course, we decided to modify the base molecule **1a** by introducing an electron-donating substituent (EDG) (case **1b**), as well as the electron-acceptor substituent (EWG) (case **1c**).

## 2. Results and Discussion

### 2.1. Electronic Properties and the Nature of Intermolecular Interactions of Cycloaddition Components

We initiated our study from the analysis of key electronic properties of the components of the HDA process. We performed this investigation based on the reactivity descriptors estimated in the framework of the Conceptual Density Functional Theory (CDFT) [23,24,25]. A similar approach was very recently used for the prediction of the local reactivity of components of many different cycloaddition processes [26,27,28,29,30,31]. As can be seen in Table 1, the electronic chemical potential of *N*-(2,2,2-trichloroethylidene)carboxamides **1a–c** is much higher than the electronic potential of dicyclohexylcarbodiimide **2**. This indicates that in the studied HDA reactions the charge should be transferred from molecule **2** to a respective reaction partner. So, in Domingo terminology [32] the analyzed cycloadditions should be treated as the Reverse Electron Density Flux (REDF) processes. According to the universal scale of the organic reactivity [33], the classification of considered addends is different. All *N*-(2,2,2-trichloroethylidene)carboxamides (**1a–c**) should be defined as strong electrophiles. For comparison, the global electrophilicity of the dicyclohexylcarbodiimide (2) does not exceed 0.5 eV. So, this component should be treated as the nucleophilic agent. Its properties are quantitatively expressed by a great value in the global nucleophilicity index (2.66 eV). It should be underlined that the difference between global electrophilicities within considered reaction pairs confirms the polar nature [34] of title reactions.

**Figure 1:**
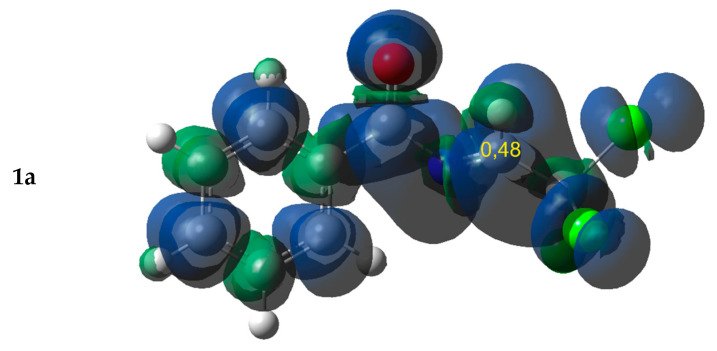

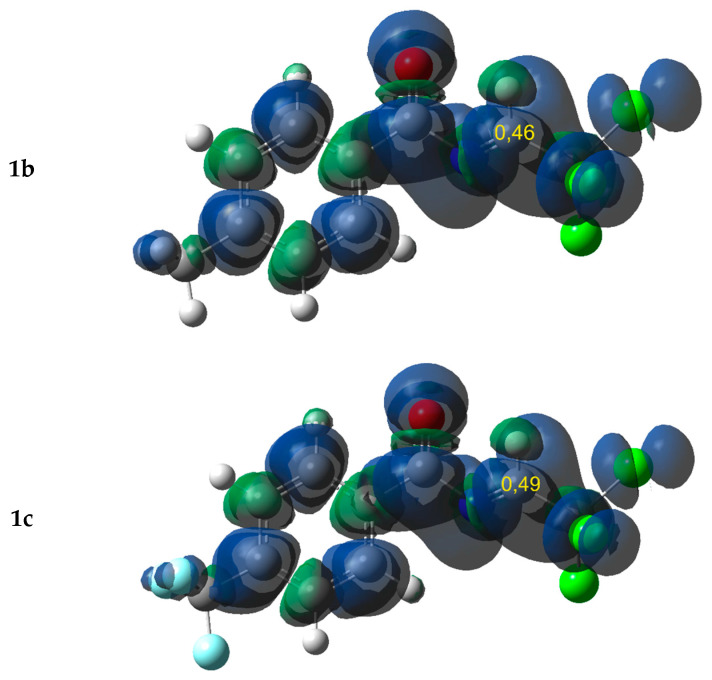
The distribution of the local electrophilicity within *N*-(2,2,2-trichloroethylidene)carboxamide (**1a**–**c**) molecules.

**Figure 2:**
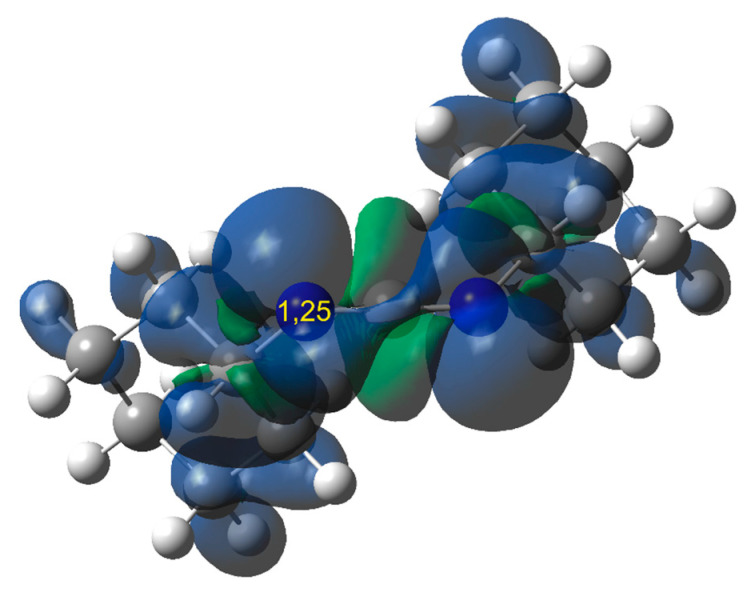
The distribution of the local electrophilicity within dicyclohexylcarbodiimide (**2**) molecule.

It is generally known [35,36,37] that within polar processes local reactivity and regioselectivity is determined by the most attractive interactions between the most activated reaction centers. In the case of considered heteroanalogues of diene, the most local electrophilicity power is localized on the C4 carbon atom of the O=C-N=C < moiety (Table 1, Figure 1). On the other hand, the most nucleophilic power within the molecule **2** is located on the nitrogen atom of the -N=C=N- moiety (Table 1, Figure 2). The mentioned interactions of most active regions of addends should lead to the formation of cycloadducts 3a–c, which excellently correlate with the experimentally observed regioselectivity.

### 2.2. Critical Points on Reaction Profiles and Critical Structures

**Figure 3:**
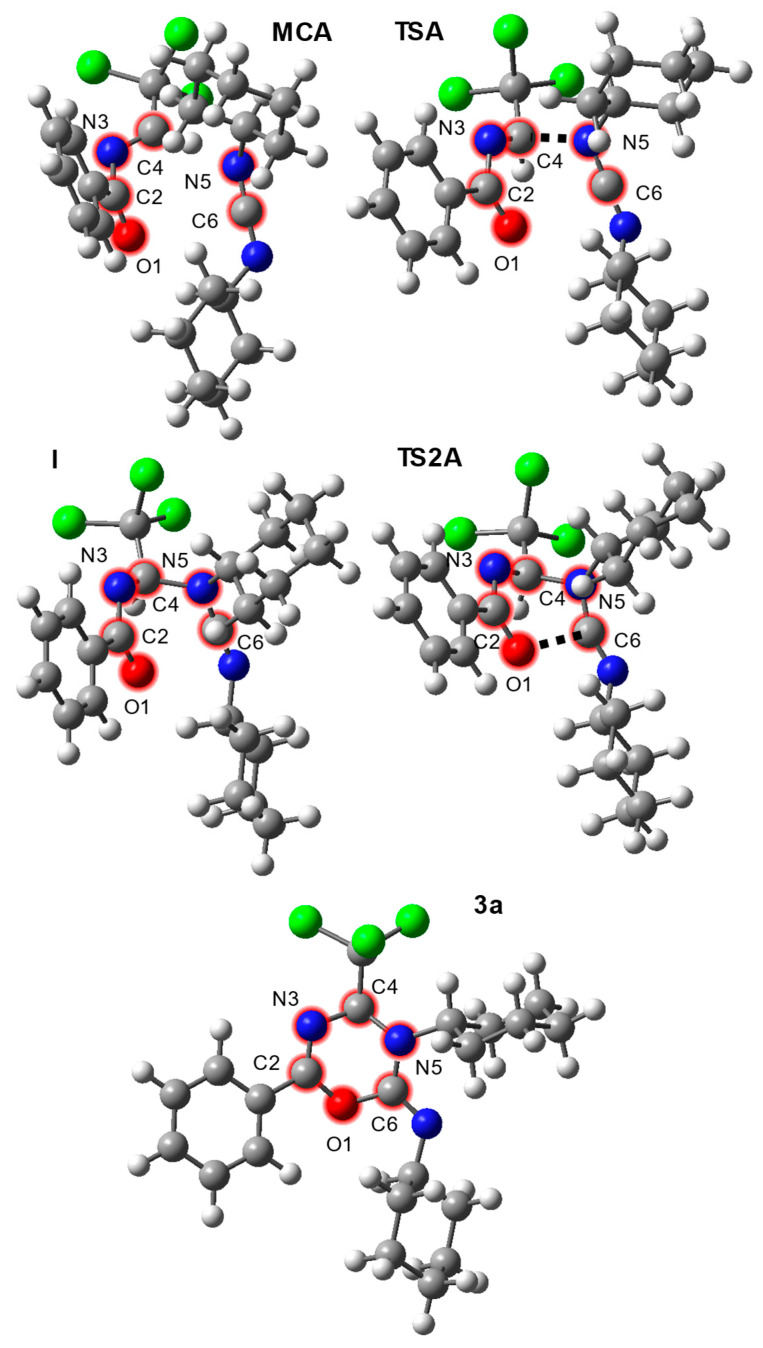
Views of critical structures of the reaction **1a + 2 → 3a** in the CCl_4_ environment in the light of the ωB97X-D/6-311G(d) (PCM) calculations.

**Figure 4:**
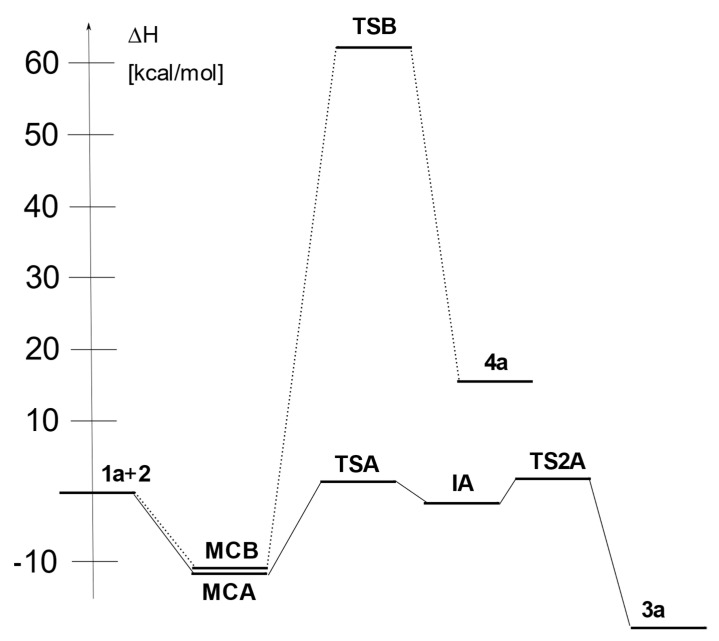
Enthalpy profiles for the [4+2] cycloaddition between *N*-(2,2,2-trichloroethylidene)carboxamide (**1a**) and dicyclohexylcarbodiimide (**2**) in the CCl_4_ environment in the light of the ωB97X-D/6-311G(d) (PCM) calculations.

**Figure 5:**
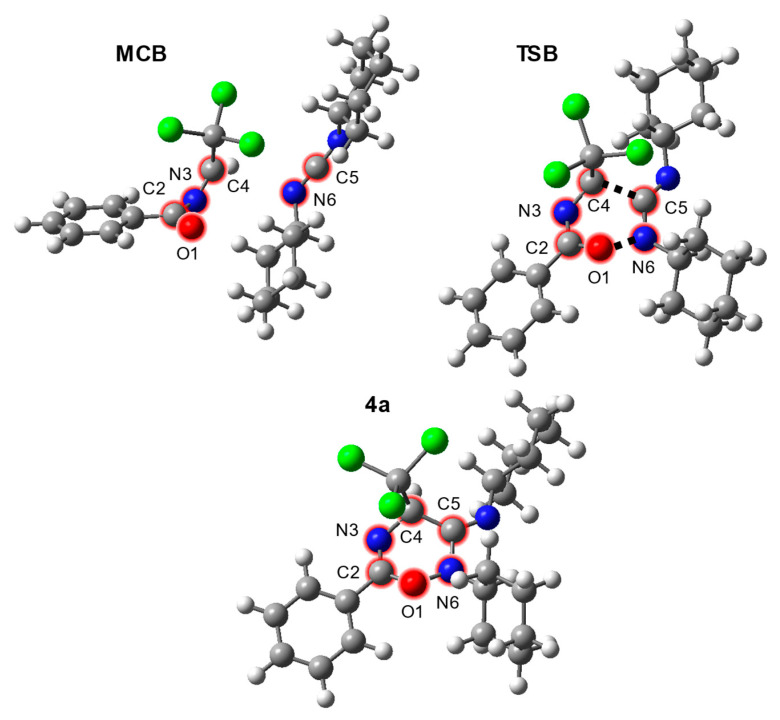
Views of critical structures of the reaction **1a + 2 → 4a** in the CCl_4_ environment in the light of the ωB97X-D/6-311G(d) (PCM) calculations.

We started the exploration of reaction profiles from the model cycloaddition **1a + 2**. According to the applied experimental conditions, a carbon tetrachloride solution was used in these calculations. It was found that the nature of the energy profiles for both competitive reaction paths are completely different. In the case of the reaction path **A** (associated with the formation of the adduct 3a), between the valleys of addends and adducts four critical points were detected and verified (Figure 3 and Figure 4). In contrast, in the case of the reaction path **B** (associated with the formation of the adduct **4a**), between valleys of addends and adducts only two critical points were detected and verified (Figure 4 and Figure 5).

In the framework of path **A**, the interactions between reaction components lead initially to the formation of the pre-reaction molecular complex **MCA** (Figure 3 and Figure 4). This process is realized as barrierless and is accompanied by the reduction of the enthalpy of the reaction system by 12.1 kcal/mol (Table 2). At the same time, the degree of order of the molecular system is substantially increased. In consequence, the formation of the **MCA** is associated with the fundamental reduction of the entropy of the reaction system. In consequence, the Gibbs free energy of the formation of the **MCA** achieves a positive value (0.9 kcal/mol). In consequence, the **MCA** cannot exist as a stable intermediate. Within the **MCA** the distances between reaction sites are beyond the range typical for new C-O and C-N sigma bonds in transition states [38,39,40]. However, reaction sites adopt the orientation, which determines the further regioisomerism of the cycloadduct (Figure 3). Thus, the localized structure can be treated as the orientation complex [41]. Analysis of the distribution of charges on reaction sites shows that the electron transfer between substructures (GEDT) is not realized at this stage. So, the considered intermediate is not the charge transfer complex. It should be mentioned at this point that a similar type of pre-reaction complexes was detected experimentally regarding the cycloaddition between ozone and ethene [42].

The further conversion of the reaction system along the reaction coordinate leads to the critical point connected with the existence of the transition state. This is the **TSA** transition state. The enthalpy of activation for this transformation is, however, very low. So, this process should be treated as kinetically allowed even at lower temperatures. In contrast to many known transition states typical for HDA reactions, within the localized TS only one new sigma bond is formed (C4-C5). At the same time, the great electron density transfer from substructure 2 to substructure 1a is observed (GEDT = 0.33 e; Table 2). This confirms the polar nature of the transition state, which was predicted based on the reactivity descriptors. The IRC calculations connect this critical point directly with the valley of the MCA and the second valley connected with the existence of the second reaction intermediate (IA) (Figure 3). Within the IA molecule, the bond C4-C5 is already fully completed. Subsequently, the great value of the GEDT index shows clearly its zwitterionic character. These types of zwitterionic intermediates were detected earlier regarding some DA and HDA processes [38,43]. To complete the formation of the target molecule 3a, the formation of the second new sigma bond C6-O1 is necessary. This process is realized in the framework of the next critical points on the reaction profile—the transition state TS2A (Figure 3). Within this structure the key distance C6-O1 is reduced up to 2.127 Å. This is a rather typical distance for the new sigma C-O bond in the transition state [44,45,46,47,48]. It should be noted that the polarity of this TS is rather high, which is confirmed by a great value in the GEDT index. The IRC calculations connect this critical point directly with the intermediate IA and with the target product 3a. From a thermodynamic point of view, the formation of this adduct is beneficial due to a negative value of the Gibbs free energy of the formation (Table 2).

The molecular mechanism is completely different for the reaction of path B. Within the initial phase, the intermolecular interactions between addends direct the reaction system to the area of the pre-reaction molecular complex **MCB** (Figure 4). This process is associated with the reduction of the enthalpy of the reaction system. This change is substantially smaller than in the case of the analogous transformation leading to the **MCA**. Within the **MCB** reaction centers adopt specific orientation, but it is a different orientation than in the case of the **MCA** intermediate (Table 3, Figure 5). These distances exist beyond the range typical for new sigma bonds in transition states. Analogously to **MCA**, **MCB** does not exhibit the nature of the charge transfer complex. The further conversion of the reaction system leads to the area of the transition state **TSB**. This transition requires very high activation energy (more than 60 kcal/mol (Table 2). So, in comparison to the activation barrier on path A, the process of **TSB** formation should be treated as forbidden from the kinetic point of view. Structurally, the TSB exhibits a completely different nature than transition states observed on path A. Within this structure, two new sigma bonds are formed. These are the C4-C5 and N6-O1 bonds. The advancement of these bonds is slightly different. Subsequently, the polarity of this transition state is relatively smaller than in the case of transition states detected on path **A**. The IRC calculations connect the **TSB** directly with the **MCB** and **4a** structures. However, it should be underlined that the transformation of the reaction system to the adduct 4a is fully unfavored due to thermodynamic factors. An extremely high Gibbs free energy in this reaction excludes the possibility of the formation of 4a along the reaction course, independently of the abovementioned kinetic factors.

In the next research stage, we also explored the influence of the polarity of solvent on the reaction course (Table 2, Table 3 and Table 4). For this purpose, the carbon tetrachloride was replaced with acetonitrile in DFT computations. It was found that in the acetonitrile solution, all activation barriers are higher by about 2 kcal/mol in comparison to the analogous process in carbon tetrachloride. These changes are not enough to reverse the reaction regioselectivity. From a mechanistic point of view, the scheme of the transformation of the molecular system along the reaction coordinate is analogous independently of the polarity of the solvent. The substituent effect on the reaction course was also explored (Table 2, Table 3 and Table 4). We detected that the influence of the nature of substituents at the aryl ring of carboxamides **1a–c** on the reaction regioselectivity and kinetic profiles is lower than in the case of the solvent effect. In consequence, the proposed regioselectivity scheme, as well as the molecular mechanism, can be treated as general for some range of HDA reaction components.

### 2.3. The ELF Study of the Reagents and BET Study of Mechanism of the Model Reaction of **1a** and **2**

**Figure 6:**
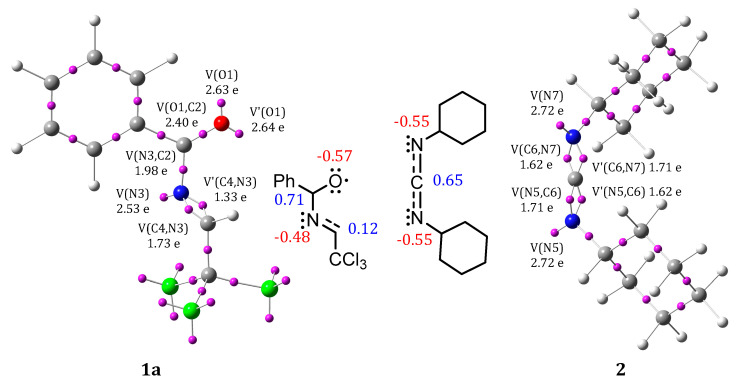
ωB97X-D/6-311G(d) ELF valence basin attractors with the most relevant valence basin populations and ELF-based Lewis-like structures with natural atomic charges of carboxamide **1a** and carbodiimide **2**. Negative charges are colored in red and positive in blue.

First, to shed some light on the electronic structure of our model reagents, carboxamide **1a** and carbodiimide **2**, we performed ELF analysis using the TopMod 09 package [49]. The obtained ELF valence basin attractors with ELF-based Lewis-like structures can be seen in Figure 6. The molecule of carboxamide 1 presents two monosynaptic basins, V(O1) and V’(O1), integrating 5.27 e which represents the nonbonding electron density of the oxygen atom of the carbonyl group. Interestingly, the disynaptic basin V(O1,C2) with a population of 2.40 e would represent a single bond in the Lewis-like structure of **1a**. On the other hand, the imine region is made up of two disynaptic basins, V(C4,N3) and V’(C4,N3), integrating 1.73 e and 1.33 e, respectively, and a monosynaptic basin V(N3) of nitrogen’s nonbonding electron density. The Natural Population Analysis (NPA) shows a significant positive charge of 0.71 e on the carbon atom of the carbonyl group, and a slightly positive charge of 0.12 e on carbon C4. The imine’s nitrogen N3 and oxygen O1 atoms exhibit a moderately negative charge of −0.48 and −0.57 e, respectively. The symmetric molecule of carbodiimide **2** consists of two disynaptic basins, V(N5,C6) and V’(C5,C6), and monosynaptic basin V(N5), integrating 1.71 e, 1.62 e, and 2.72 e. The mirrored basins V(C6,N7), V’(C6,N7) and V(N7) show identical populations. The NPA revealed a positive charge of 0.65 e on the central carbon C6 and negative charges of −0.55 e on the nitrogen atoms N5 and N7.

In the next step, we studied the mechanism of the model reaction of carboxamide **1a** with carbodiimide **2** using bonding evolution theory (BET) analysis [50].

**Figure 7:**
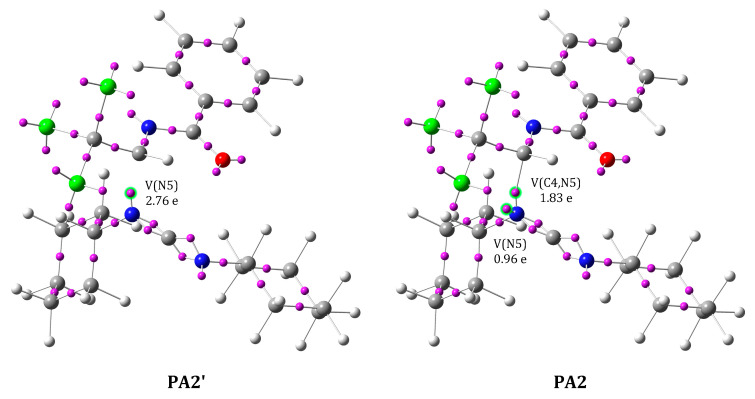
ELF valence basin attractors of structures of the most important topological changes, creation of C4-N5 bond, during the reaction of carboxamide **1a** and carbodiimide **2** along the reaction path leading to zwitterion IA. Structures with apostrophe represent the first structure before the critical point.

**Scheme 5:**
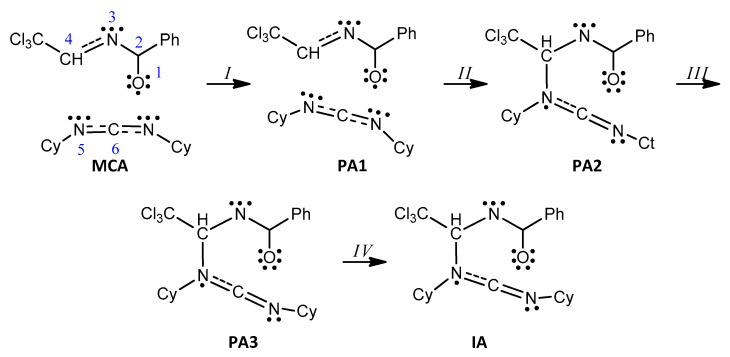
Simplified representation of the molecular mechanism of the addition of carboxamide **1a** and carbodiimide **2** by Lewis-like structures based on the topological analysis of the ELF along the reaction path leading to zwitterion IA.

The addition of carbodiimide **2** to carboxamide **1a** studied with BET revealed four phases in its reaction path; the critical points of this path are named PA. The BET data is collected in Table 5, and the ELF-based Lewis-like structures are depicted in Scheme 5. The molecular complex shows only minimal changes in electron density compared to separate reagents **1a** and **2**. The first significant change takes place in PA1 where two disynaptic basins, V(C4,N3) and V’(C4,N3), merge into one V(C4,N3), integrating 2.95 e. The GEDT in PA1 is only 0.01 e, but the transition state TSA located in this phase exhibits a significantly increased GEDT by 0.35 e. The III phase, *d*(C4-N5) = 1.796 Å, starts with the creation of a new bond C4-N5 by donation of a part of the nonbonding electron density of N5, represented by monosynaptic basin V(N5), to carbon atom C4. The newly created disynaptic basin V(C4,N5) shows a population of 1.83 e, where integration of V(N5) decreases to 0.96 e; the structure PA2 and the directly preceding PA2′ can be seen in Figure 7. The last phase starts at PA3 where a merging of two disynaptic basins, V(N5,C6) and V’(N5,C6), of the carbodiimide region into one V(N5,C6) can be observed. The final structure of this phase, which is the intermediate zwitterion IA, exhibits a slight increase in the nonbonding electron densities of atoms O1, N3 and N5. On the other hand, the disynaptic basins V(O1,C2), V(C4,N3) and V(N5,C6) show a decrease in population, interestingly, as well as V(C4,N5) of the newly created bond C4-N5. These changes are in line with thhe electron density distribution of cycloadduct **3a**.

**Scheme 6:**
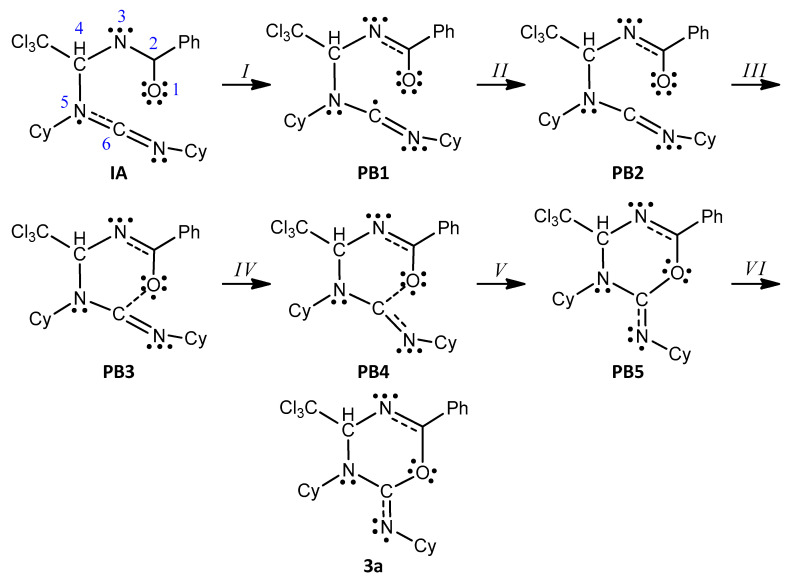
Simplified representation of the molecular mechanism of cyclization of zwitterion IA by Lewis-like structures based on the topological analysis of the ELF along the reaction path leading to product 3a.

**Figure 8:**
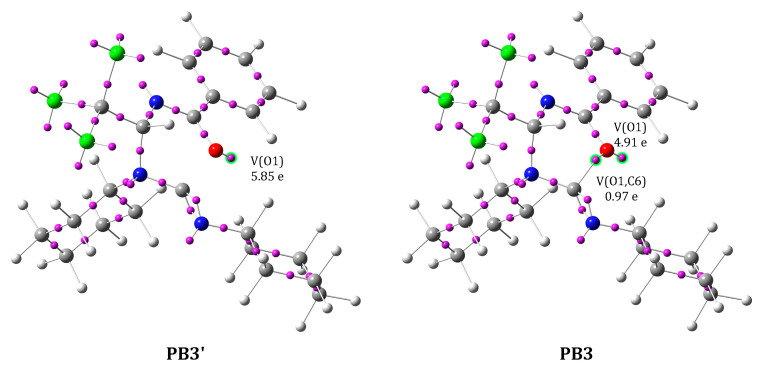
ELF valence basin attractors of structures of the most important topological changes, creation of O1-C6 bond, during cyclization of zwitterion **IA** along the reaction path leading to product 3a. Structures with apostrophe represent the first structure before the critical point.

The second part of the reaction of carboxamide **1a** with carbodiimide **2** proceeds by cyclization of zwitterion **IA** in six phases based on the BET analysis; the critical points of this path are named PB. The BET data is collected in Table 6, and the ELF-based Lewis-like structures are depicted in Scheme 6. During the first phase a rearrangement of electron density takes place, in particular the disynaptic basins V(O1,C2) and V(N5,C6) decrease in population and at the same time the disynaptic basin V(N3,C2) and monosynaptic basins V(N5) and V(N7) show an increase. In this phase the transition state TS2A is located and a GEDT between carboxamide and carbodiimide fragments of the structure starts to decrease. Point PB2 begins phase II with the creation of a new monosynaptic basin V(C6) integrating 0.04 e, and the disappearance of V’(O1), with its population transferred to V(O1). At the next phase the basin V(C6) is destroyed, and at the same time the population of basin V(O1) increases to 5.84 e. The GEDT decreases to 0.31 e. Point PB3, *d*(O1-C6) = 1.703 Å and *d*(C4-N5) = 1.474 Å, marks the creation of a second new bond O1-C6 by donation of the nonbonding electron density of O1, represented by monosynaptic basin V(O1), to C6. The newly created disynaptic basin V(O1,C6) integrated 0.97 e, while the population of V(O1) decreased to 4.91 e. The point PB3 at which the new bond is established and directly preceding point PB3′ is shown on Figure 8. Phase V starts with a split of disynaptic basin V(N3,C2) into two, V(N3,C2) and V’N3,C2), integrating 1.42 and 1.50, respectively. The V(O1,C6) basin of the new bond increases in population to 1.09 e. The last phase begins with the creation of a second monosynaptic basin V’(N5). In the meantime, the V(O1) basin’s integration decreases to 4.49 e, and the GEDT lowers to 0.14 e. In the product 3a, a further increase in the population of monosynaptic basins V’(N5) and V(N7) was observed, while integrations of V(N5), V(C6,N7) and V’(C6,N7) decreased.

## 3. Computational Details

The explorations of the reaction profiles were performed based on the quantum-chemical DFT calculations. The ωB97X-D/6-311G(d) level of theory from the Gaussian 09 software package [51] was used. Transition states were localized using the QST2 procedure. At the same time, an alternative approach was applied for localization of transition states on reaction paths leading to adducts 3 and 4. In particular, the molecular systems were scanned with a gradual reduction of key interatomic distances. Using both different ways we obtained the same sequence of critical structures along the reaction path, for the **A** and **B** cycloaddition channels. All attempts at searching for the alternative mechanisms of reactions **A** and **B** were not successful.

All localized and optimized stationary points were characterized using vibrational analysis. It was found that starting molecules, intermediates and products had positive Hessian matrices. On the other hand, all transition states (TS) showed only one negative eigenvalue in their Hessian matrices. The calculations were performed for T = 298 K.

Intrinsic reaction coordinate (IRC) calculations were performed for the verification of all optimized transition states. The presence of the solvent in the reaction environment (tetrachloromethane, acetonitrile) was included using the IEFPCM algorithm [52].

The global electron density transfer (GEDT) [53] within critical structures (MCs and TSs) was estimated using the following formula:

GEDT = −Σ*q*A (1)

where *q*A is the net charge, and the sum is taken over all the atoms of the substructure of the nitroalkene.

The same level of theory was used within the ELF analysis

Global and local electronic properties of the reactants were estimated according to the equations recommended earlier by Parr and Domingo [54,55,56]. According to Domingo’s recommendation, for this purpose the ωB97X-D/6-311G(d) level of theory was used. All molecules were fully optimized. Next, electronic chemical potentials (*μ*) and chemical hardness (*η*) were evaluated in terms of one-electron energies of FMO (Ε_HOMO_ andΕ_LUMO_) using the following equations:

*μ* ≈ (E_HOMO_ + E_LUMO_)/2 η ≈ E_LUMO_ − E_HOMO_ (2)

Next, the values of *µ* and *η* were then used to calculate the global electrophilicity index (*ω*) according to the following formula:

*ω* = *μ*^2^/2*η* (3)

Subsequently, global nucleophilicity (N) [57] can be expressed in terms of the equation (E_HOMO (tetracyanoethene)_ (11.4 eV) and was calculated using the ωB97xd/6-311G(d) level of theory):

*N* = E_HOMO_ − E_HOMO (tetracyanoethene)_ (4)

The local electrophilicity (*ω*_k_) condensed to atom *k* was calculated by projecting the index *ω* onto any reaction center *k* in the radical cation of the molecule using Parr functions *P*^+^_k_ [58] represented by Mulliken spin densities:

*ω*_k_ = *P*^+^_k_·*ω* (5)

The local nucleophilicity (*N*_k_) condensed to atom *k* in the radical anion of the molecule was calculated using global nucleophilicity *N* and Parr functions *P*^-^_k_ [58] according to the following formula:

*N*_k_ = *P*^−^_k_·*N* (6)

## 4. Conclusions

This ωB97xd/6-311G(d) (PCM) computational study offers a comprehensive view on factors that determine the global and local reactivity of the components of the Diels–Alder reactions between *N*-(2,2,2-trichloroethylidene)carboxamides and dicyclohexylcarbodiimide. It was found that the considered processes should be treated as polar and determined by the local interaction between the C4 carbon atom of the O=C-N=C< moiety of the heteroanalogue of diene and the nitrogen atom of the -N=C=N- moiety of the dicyclohexylcarbodiimide. The polar nature of the title reactions is strong, which determines the stepwise cycloaddition mechanism with the intervention of the zwitterionic intermediate on a kinetically favored path. It should be underlined, that the competitive reaction path that leads to the regioisomeric adduct should be treated as forbidden from a kinetic point of view and not beneficial from a thermodynamic point of view. The details of the electron density redistribution along the reaction coordinate can be explained using the ELF technique.

## Figures and Tables

**Table 1:** Global and local electronic properties of *N*-(2,2,2-trichloroethylidene)carboxamides (**1a**–**c**) and dicyclohexylcarbodiimide (**2**).

	Global Properties				Local Properties 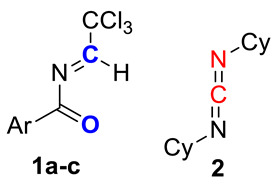							
	*μ* [eV]	*η* [eV]	*ω* [eV]	*N* [eV]	*P* ^−^_N_	*P* ^−^_C_	*N*_N_ [eV]	*N*_C_ [eV]	*P* ^+^_C_	*P* ^+^_O_	*ω*_C_ [eV]	*ω*_O_ [eV]
**1a**	−5.06	8.86	1.45	1.91					0.333	0.176	0.48	0.25
**1b**	−4.87	8.61	1.38	2.23					0.335	0.175	0.46	0.24
**1c**	−5.49	8.98	1.68	1.42					0.295	0.197	0.49	0.33
**2**	−3.05	11.38	0.41	2.66	0.473	−0.163	1.25	−0.43				

**Table 2:** Kinetic and thermodynamic parameters for the [4+2] cycloadditions between *N*-(2,2,2-trichloroethylidene)carboxamides (**1a–c**) and dicyclohexylcarbodiimide (**2**) in the light of the ωB97X-D/6-311G(d) (PCM) calculations (ΔH, ΔG are in kcal/mol; ΔS are in cal/molK).

Solvent	Path	Transition	ΔH	ΔS	ΔG
CCl_4_	**A**	**1a + 2 → MCA**	−12.1	−43.8	0.9
		**1a + 2 → TSA**	1.5	−54.0	17.6
		**1a + 2 → IA**	−1.6	−55.2	14.8
		**1a + 2 → TS2A**	−0.9	−61.8	17.5
		**1a + 2 → 3a**	−20.4	−63.0	−1.6
	**B**	**1a + 2 → MCB**	−4.9	−43.5	8.0
		**1a + 2 → TSB**	62.6	−58.4	79.9
		**1a + 2 → 4a**	15.4	−61.4	33.7
MeCN	**A**	**1a + 2 → MCA**	−10.9	−45.1	2.5
		**1a + 2 → TSA**	2.7	−53.8	18.7
		**1a + 2 → IA**	−2.0	−54.2	14.2
		**1a + 2 → TS2A**	−0.1	−62.7	18.6
		**1a + 2 → 3a**	−18.2	−63.9	0.8
	**B**	**1b + 2 → MCB**	−4.6	−47.0	9.4
		**1b + 2 → TSB**	64.1	−59.5	81.8
		**1b + 2 → 4a**	17.2	−61.8	35.6
CCl_4_	**A**	**1b + 2 → MCA**	−12.3	−46.1	1.4
		**1b + 2 → TSA**	0.4	−52.2	15.9
		**1b + 2 → IA**	−1.5	−62.4	17.1
		**1b + 2 → TS2A**	−0.5	−59.0	17.1
		**1b + 2 → 3b**	−20.3	−61.5	−2.0
	**B**	**1b + 2 → MCB**	−5.4	−47.5	8.8
		**1b + 2 → TSB**	62.7	−58.5	80.2
		**1b + 2 → 4b**	15.6	−60.4	33.6
CCl_4_	**A**	**1c + 2 → MCA**	−13.2	−46.5	0.7
		**1c + 2 → TSA**	−2.2	−62.1	16.4
		**1c + 2 → IA**	−3.9	−55.8	12.8
		**1c + 2 → TS2A**	−2.7	−61.0	15.4
		**1c + 2 → 3c**	−20.8	−62.3	−2.2
	**B**	**1c + 2 → MCB**	−5.7	−49.2	9.0
		**1c + 2 → TSB**	61.4	−55.2	77.9
		**1c + 2 → 4c**	14.4	−69.5	35.2

**Table 3:** Key parameters for critical structures of the reactions **1a−c + 2 → 3a−c** in the light of the ωB97X-D/6-311G(d) (PCM) calculations.

Solvent	Reaction	Structure	Interatomic Distances [Å]						GEDT [e]
			O1-C2	C2-N3	N3-C4	C4-N5	N5-C6	C6-O1	
CCl_4_	**1a** + **2**	**1a**					1.217		
		**2**	1.206	1.420	1.253				
		**MCA**	1.207	1.410	1.254	3.207	1.221	3.481	
		**TSA**	1.233	1.365	1.318	1.867	1.244	2.837	−0.33
		**IA**	1.254	1.334	1.385	1.548	1.269	2.625	−0.58
		**TS2A**	1.274	1.316	1.404	1.509	1.294	2.127	−0.51
		**3a**	1.357	1.262	1.441	1.468	1.376	1.389	
MeCN	**1a** + **2**	**1a**					1.218		
		**2**	1.208	1.413	1.252				
		**MCA**	1.209	1.410	1.254	3.209	1.219	3.503	
		**TSA**	1.231	1.370	1.314	1.908	1.242	2.910	−0.32
		**IA**	1.252	1.334	1.392	1.532	1.271	2.765	−0.63
		**TS2A**	1.278	1.314	1.411	1.499	1.301	2.065	−0.52
		**3a**	1.356	1.263	1.442	1.469	1.376	1.389	
CCl_4_	**1b** + **2**	**1b**					1.217		
		**MCA**	1.208	1.411	1.254	3.236	1.221	3.519	
		**TSA**	1.233	1.371	1.317	1.860	1.244	2.938	−0.34
		**IA**	1.254	1.335	1.384	1.549	1.269	2.622	−0.57
		**TS2A**	1.274	1.317	1.403	1.510	1.294	2.134	−0.51
		**3b**	1.359	1.262	1.441	1.468	1.376	1.389	
CCl_4_	**1c** + **2**	**1c**					1.217		
		**MCA**	1.206	1.406	1.254	3.221	1.221	3.526	
		**TSA**	1.229	1.371	1.313	1.905	1.242	2.985	−0.32
		**IA**	1.252	1.332	1.388	1.542	1.269	2.649	−0.59
		**TS2A**	1.275	1.312	1.408	1.504	1.298	2.094	−0.51
		**3c**	1.354	1.260	1.442	1.468	1.377	1.391	

**Table 4:** Key parameters for critical structures of the reactions **1a−c + 2 → 4a−c** in the light of the ωB97X-D/6-311G(d) (PCM) calculations.

Solvent	Reaction	Structure	Interatomic Distances [Å]						GEDT [e]
			O1-C2	C2-N3	N3-C4	C4-C5	C5-N6	N6-O1	
CCl_4_	**1a** + **2**	**MCB**	1.210	1.401	1.251	3.785	1.219	5.086	
		**TSB**	1.251	1.341	1.338	1.880	1.288	2.128	−0.22
		**4a**	1.349	1.270	1.444	1.531	1.397	1.413	
MeCN	**1a** + **2**	**MCB**	1.212	1.398	1.251	3.819	1.219	5.215	
		**TSB**	1.250	1.342	1.340	1.878	1.289	2.138	−0.23
		**4a**	1.348	1.271	1.446	1.531	1.397	1.413	
CCl_4_	**1b** + **2**	**MCB**	1.210	1.402	1.251	3.791	1.219	5.083	
		**TSB**	1.251	1.342	1.338	1.879	1.288	2.127	−0.21
		**4b**	1.350	1.271	1.444	1.531	1.396	1.412	
CCl_4_	**1c** + **2**	**MCB**	1.208	1.397	1.252	3.780	1.219	5.062	
		**TSB**	1.249	1.339	1.340	1.877	1.289	2.138	−0.24
		**4c**	1.347	1.269	1.445	1.531	1.399	1.414	

**Table 5:** ELF valence basin populations of the IRC points, MCA—zwitterion IA defining the four different phases characterizing the reaction of the carboxamide **1a** and carbodiimide **2**. Stationary points **1a**, **2**, and **TSA** are also included. Distances are given in angstroms, Å, electron populations in an average number of electrons, [e], relative energies in kcal·mol^−1^, GEDT values in an average number of electrons, [e].

Structures	1a	2	MCA	PA1	TSA	PA2	PA3	IA
**Phases**	-	-	I	II		III	IV	-
*d*(C4-N5)	-	-	3.207	2.872	1.867	1.796	1.738	1.548
GEDT	-	-	0.01	0.01	0.35	0.41	0.45	0.56
*d*E	-	-	−13.62	−8.43	0.42	0.27	−0.09	−3.96
V(O1)	2.63	-	2.67	2.63	2.70	2.71	2.73	2.74
V’(O1)	2.64	-	2.61	2.65	2.77	2.79	2.82	2.90
V(O1,C2)	2.40	-	2.39	2.40	2.20	2.16	2.13	2.01
V(N3,C2)	1.98	-	2.00	2.00	2.23	2.27	2.32	2.44
V(N3)	2.53	-	2.52	2.64	3.12	3.19	3.23	3.33
V(C4,N3)	1.73	-	1.66	2.95	2.36	2.24	2.15	1.93
V’(C4,N3)	1.33	-	1.39	-	-	-	-	-
V(N5,C6)	-	1.71	1.68	1.68	1.76	1.80	3.18	2.99
V’(N5,C6)	-	1.62	1.61	1.63	1.39	1.33	-	-
V(N5)	-	2.72	2.78	2.74	2.74	0.96	1.08	1.35
V(C6,N7)	-	1.62	1.60	1.54	1.73	1.75	1.78	1.90
V’(C6,N7)	-	1.71	1.73	1.78	1.88	1.89	1.91	1.93
V(N7)	-	2.72	2.73	2.76	2.44	2.41	2.38	2.22
V(C4,N5)	-	-	-	-	-	1.83	1.66	1.60

**Table 6:** ELF valence basin populations of the IRC points, zwitterion **IA**—product **3a**, defining the six different phases characterizing the cyclization reaction of the zwitterion **IA**. Stationary point **TS2A** is also included. Distances are given in angstroms, Å, electron populations in an average number of electrons, [e], relative energies in kcal·mol^−1^, GEDT values in an average number of electrons, [e].

Structures	IA	TS2A	PB1	PB2	PB3	PB4	PB5	3a
**Phases**	I		II	III	IV	V	VI	-
*d*1(C4-N5)	1.548	1.509	1.486	1.478	1.474	1.469	1.455	1.468
*d*2(O1-C6)	2.625	2.127	1.865	1.756	1.703	1.631	1.406	1.389
GEDT	0.56	0.49	0.37	0.31	0.28	0.25	0.14	0.11
*d*E	0.00	1.2	−0.5	−2.7	−4.1	−6.3	−16.7	−19.7
V(O1)	2.74	2.49	5.62	5.84	4.91	4.83	4.49	4.46
V’(O1)	2.90	3.15	-	-	-	-	-	-
V(O1,C2)	2.01	1.89	1.77	1.70	1.68	1.64	1.59	1.58
V(N3,C2)	2.44	2.60	2.77	2.84	2.88	1.42	1.52	1.54
V’(N3,C2)	-	-	-	-	-	1.50	1.50	1.52
V(N3)	3.33	3.22	3.09	3.03	3.00	2.95	2.89	2.85
V(C4,N3)	1.93	1.88	1.85	1.84	1.84	1.83	1.79	1.79
V(N5,C6)	2.99	2.67	2.40	2.22	2.16	2.11	2.04	2.07
V(N5)	1.35	1.64	1.85	1.92	1.96	2.00	1.98	1.58
V(C6,N7)	1.90	1.93	1.88	1.83	1.81	1.78	1.71	1.68
V’(C6,N7)	1.93	1.81	1.78	1.73	1.70	1.69	1.61	1.53
V(N7)	2.22	2.35	2.50	2.56	2.59	2.63	2.75	2.86
V(C4,N5)	1.60	1.67	1.70	1.71	1.72	1.72	1.75	1.65
V(O1,C6)	-	-	-	-	0.97	1.09	1.50	1.55
V’(N5)	-	-	-	-	-	-	0.27	0.73
V(C6)	-	-	0.04	-	-	-	-	-

## Data Availability

The original contributions presented in this study are included in the article. Further inquiries can be directed to the corresponding author(s).
